# Alzheimer’s disease protein relevance analysis using human and mouse model proteomics data

**DOI:** 10.3389/fsysb.2023.1085577

**Published:** 2023-07-13

**Authors:** Cathy Shi, W. Kirby Gottschalk, Carol A. Colton, Sayan Mukherjee, Michael W. Lutz

**Affiliations:** ^1^ Department of Statistical Science, Duke University, Durham, NC, United States; ^2^ Division of Translational Brain Sciences, Department of Neurology, Duke University School of Medicine, Durham, NC, United States; ^3^ Departments of Mathematics, Computer Science, and Biostatistics and Bioinformatics Duke University, Durham, NC, United States

**Keywords:** Alzheimer’s disease proteomics analysis, Alzheimer’s disease proteomics statistics, Alzheimer’s disease mouse models, Alzheimer’s disease proteomics: human and mouse comparisons, extracellular matrix proteins and Alzheimer’s disease, integrin related pathways and Alzheimer’s disease

## Abstract

The principles governing genotype-phenotype relationships are still emerging (Jovanovic, Science, 2015, 347 (6,226), 1,259,038; Buccitelli et al., Nature Reviews Genetics, 2020, 21 (10), 630–44; Öztürk et al., Nature Communications, 2022, 131), 6,153), and detailed translational as well as transcriptomic information is required to understand complex phenotypes, such as the pathogenesis of Alzheimer’s disease. For this reason, the proteomics of Alzheimer disease (AD) continues to be studied extensively. Although comparisons between data obtained from humans and mouse models have been reported, approaches that specifically address the between-species statistical comparisons are understudied. Our study investigated the performance of two statistical methods for identification of proteins and biological pathways associated with Alzheimer’s disease for cross-species comparisons, taking specific data analysis challenges into account, including collinearity, dimensionality reduction and cross-species protein matching. We used a human dataset from a well-characterized cohort followed for over 22 years with proteomic data available. For the mouse model, we generated proteomic data from whole brains of CVN-AD and matching control mouse models. We used these analyses to determine the reliability of a mouse model to forecast significant proteomic-based pathological changes in the brain that may mimic pathology in human Alzheimer’s disease. Compared with LASSO regression, partial least squares discriminant analysis provided better statistical performance for the proteomics analysis. The major biological finding of the study was that extracellular matrix proteins and integrin-related pathways were dysregulated in both the human and mouse data. This approach may help inform the development of mouse models that are more relevant to the study of human late-onset Alzheimer’s disease.

## 1 Introduction

Genome-wide association studies and multiple -omics studies, including proteomics, have revealed that AD pathology is accompanied by perturbations in multiple metabolic and biological pathways that impacts virtually all cell types in the brain (Thompson et al., 2003; Wan et al., 2020a).

Because each protein represents one structural gene, the extensive information represented in proteomics datasets, can be analyzed by appropriate statistical methods to identify structural genes and pathways that are highly correlated with AD, and hence, provide a focus for further research. As is the case for most late-onset neurodegenerative diseases, for Alzheimer’s Disease there are specific aspects of protein dysmetabolism, specifically misfolding and aggregation of specific proteins into abnormal, toxic species that define the neuropathology. Recent work has supported the premise that many biological changes relevant to AD pathophysiology are occurring through mechanisms that are not reflected through changes in mRNA abundance or co-expression (Johnson et al., 2022). This work emphasizes the importance of proteomics analysis and the integration of multiple levels of omics data for understanding the biological mechanisms that underlie development of AD.

Prior multilayer brain proteomic and phosphoproteomic studies have identified molecular networks, including amyloid cascade, inflammation, complement, WNT signaling, TGF-β, BMP signaling, lipid metabolism, iron homeostasis ad membrane transport that are involved in AD progression and contrasted molecular signatures in brain tissue and cerebrospinal fluid proteomic (CSF) with the 5xFAD mouse model (Bai et al., 2020). Reviews of proteomics methods and analysis strategies for unbiased deep profiling of the proteome, specifically differentially expressed proteins and post-translational modifications associated with Alzheimer’s disease have been published (Bai et al., 2021). Multiplexed tandem-mass-tag for ultra-deep proteomics coverage followed by systems biology analysis revealed specific protein signatures for AD across the cortex, CSF and serum that highlighted mitochondrial proteins as involved with the development of AD (9).

For this study, we used human proteomic data from the ROSMAP [Religious Orders Study and the Memory and Aging Project]) study that contains a cohort of 387 individuals well-characterized in terms of sex, race, education, and their state of AD development (Bennett et al., 2012a; Bennett et al., 2012b; Bennett et al., 2018). This dataset contains measurements of 4,913 proteins from the dorsolateral cortex of each individual enrolled in the study.

The mouse proteomic data were obtained from whole brain samples of the CVN-AD AD mouse model. This model faithfully recapitulates the three primary pathologies of human Alzheimer’s disease, amyloid deposits, the accumulation of neurofibrillary tangles, and neuron loss, with minimal genetic manipulation. It is a transgenic model that incorporates human APP bearing the Swedish/Dutch/Iowa (APPSwDI) amyloidogenic mutations under control of the Thy1 promoter (Davis et al., 2004; Wilcock et al., 2008; Colton CA. et al., 2014), on the Nos2 knock-out background. Unlike many other mouse AD models used up until now, the introduced human APP is expressed at a low level, only ∼0.5X the level of endogenous App (Davis et al., 2004). We placed this mutation on the Nos2 knock-out background because inducible nitric oxide synthase has a key role in innate immunity (Bogdan, 2015), and the innate immune response is critical for both the initiation and progression of AD ((Zhang et al., 2013; Kan et al., 2015; Shi and Holtzman, 2018)), However, the expression and activity of human NOS2 are significantly lower than for the mouse Nos2 ((Colton et al., 1996; Mestas and Hughes, 2004)); in order to mimic the human condition we knocked-out Nos2 expression. APPSwDI mice display only amyloid pathology (Davis et al., 2004), and the Nos2 knock-out mice do not exhibit any AD pathology. By contrast, the APPSwDI/Nos2^−/−^ (CVN-AD) mice develop amyloid plaques and tau pathology, including hyperphosphorylated tau and the accumulation of neurofibrillary tangles, and exhibit neuron loss and learning and memory deficits reminiscent of human AD (Wilcock et al., 2008; Colton C. et al., 2014). Control studies showed that CVN-AD mice exhibit the same pathologies as APPSwDI/huNOS2^Tg^, representing CVN-AD engineered to express human NOS2 (Colton CA. et al., 2014). Knocking out the endogenous Nos2 therefore faithfully phenocopies the consequences of the human gene. We have also confirmed the effects of knocking out Nos2 on tau pathology, by crossing another amyloid model, Tg2576 (APPSw), with Nos2 knock-out mice (Colton et al., 2006). Because limited genetic changes, based on well-known and established biology, elicit AD pathology, we chose the CVN-AD model for the studies reported in this paper. The mouse model proteome dataset contains expression measurements of 2014 proteins in 40 samples, and covariate information including mouse model genotype, sex and age.

Regression models have been useful for the analysis of proteomics data. However, the type of regression models must be carefully selected based on their functionalities and advantages in overcoming the challenges of high dimensionality and co-linearity present in the proteomics data. In addition to the problem of high dimensionality, in which the number of proteins (p) far exceeds the number of observations (n), collinearity in the feature space is also a critical issue since expression levels of many related proteins are highly correlated. In this study, we contrast LASSO regression with partial least squares-discriminant analysis (PLS-DA), a variant of Partial Least Squares Regression (PLSR). We compared the mouse and human proteomic analyses at the individual protein and biochemical pathway levels.

## 2 Methods

### 2.1 Description of datasets used

#### 2.1.1 Human data

The human data sample was taken from a subset of the Religious Orders Study and Rush Memory and Aging Project (ROSMAP) dataset (Bennett et al., 2012a; Bennett et al., 2012b; De Jager et al., 2014) that had proteomics data available from the dorsolateral frontal cortex. ROS has enlisted nuns and brothers since 1994. MAP recruited individuals from the northern Illinois region since 1997. Both studies were run by the same investigators using similar data collection techniques. Thus, the results from both were comparable. For the analyses reported in this paper, the clinical consensus diagnoses of Alzheimer’s disease or mild cognitive impairment were used to define a case while the diagnosis of no cognitive impairment/no impaired domains defined controls. Additional covariates for the statistical models were age, sex and APOE genotype. The total sample with proteomics data contained 387 subjects, with 221 cases and 166 controls. Demographic information for the sample is summarized in Table 1. Data for the human samples was generated from tandem-mass-tag proteomics (TMT). A complete description of the tissue preparation and mass spectrometry is given in Johnson et al. (Johnson et al., 2020) and described on the data description page available in the Alzheimer’s Disease Knowledge Portal (https://www.synapse.org/#!Synapse:syn17015098). In brief, before TMT labeling, individuals were randomized by covariates (such as age, sex, PMI and diagnosis), into 50 total batches (eight individuals per batch). Peptides from each individual (*n* = 400) and the GIS pooled standard (*n* = 100) were labeled using the TMT 10-plex kit (Thermo Fisher Scientific, 90,406). Labeling was performed as described in Johnson et al. (Johnson et al., 2018) and Ping et al. (Ping et al., 2018).

**TABLE 1:** (Panel A) Human sample demographics. (Panel B) Mouse sample demographics.

Status	Sex	N	Age (years) mean (SD)
AD	Female	156	88.6 (2.90)
AD	Male	65	86.6 (3.99)
NCI	Female	116	86.9 (4.32)
NCI	Male	50	85.5 (5.04)

Genotype	Sex	N	Age (months) mean (SD)
ApoE-3	F	3	54.0 (2.65)
ApoE-3	M	2	66.0 (7.07)
ApoE-4	F	3	55.7 (11.68)
ApoE-4	M	2	50.5 (10.61)
CVN-AD	F	3	54.0 (1.73)
CVN-AD	M	3	52.0 (1.00)
E3 HN	F	3	59.0 (0.00)
E3 HN	M	3	55.0 (0.00)
E4HN	F	3	68.0 (0.00)
E4HN	M	3	58.3 (2.89)
HuNOS2	F	3	54.7 (1.15)
HuNOS2	M	3	52.7 (10.97)
NOS2 KO	F	3	62.3 (0.58)
NOS2 KO	M	3	56.7 (4.62)

##### 2.1.1.1 High-pH off-line fractionation of brain tissues (50 10-plex TMT batches)

High pH fractionation was performed essentially as described in Ping et al. (Ping et al., 2020) with slight modification. Dried peptide samples were resuspended in high-pH loading buffer (0.07% vol/vol NH4OH, 0.045% vol/vol FA, 2% vol/vol ACN) and loaded onto an Agilent ZORBAX 300 Extend-C18 column (2.1 × 150 mm with 3.5 µm beads). An Agilent 1100 HPLC system was used to carry out fractionation. Solvent A consisted of 0.0175% (vol/vol) NH4OH, 0.01125% (vol/vol) FA and 2% (vol/vol) ACN; solvent B consisted of 0.0175% (vol/vol) NH4OH, 0.01125% (vol/vol) FA and 90% (vol/vol) ACN. The sample elution was performed over a 58.6-min gradient with a flow rate of 0.4 ml min−1. The gradient consisted of 100% solvent A for 2 min, then 0%–12% solvent B over 6 min, then 12% to 40% over 28 min, then 40%–44% over 4 min, then 44%–60% over 5 min and then held constant at 60% solvent B for 13.6 min. A total of 96 individual equal volume fractions were collected across the gradient and subsequently pooled by concatenation into 24 fractions and dried to completeness using a SpeedVac.

##### 2.1.1.2 TMT-MS of brain tissues

All fractions were resuspended in an equal volume of loading buffer (0.1% FA, 0.03% TFA, 1% ACN) and analyzed by liquid chromatography coupled to tandem MS essentially as described, with slight modifications. Peptide eluents were separated on a self-packed C18 (1.9 μm) fused silica column (25 cm × 75 μM internal diameter; New Objective) by an Dionex UltiMate 3,000 RSLCnano liquid chromatography system (Thermo Fisher Scientific) and monitored on an Orbitrap Fusion mass spectrometer (Thermo Fisher Scientific). Sample elution was performed over a 180-min gradient with flow rate at 225 nL min−1. The gradient was from 3% to 7% buffer B over 5 min, then 7%–30% over 140 min, then 30%–60% over 5 min, then 60%–99% over 2 min, then held constantly at 99% solvent B for 8 min and then back to 1% B for an additional 20 min to equilibrate the column. Buffer A was water with 0.1% (vol/vol) FA and buffer B was 80% (vol/vol) acetonitrile in water with 0.1% (vol/vol) FA. The mass spectrometer was set to acquire in data-dependent mode using the top speed workflow with a cycle time of 3 s. Each cycle consisted of one full scan followed by as many MS/MS (MS2) scans that could fit within the time window. The full scan (MS1) was performed with an m/z range of 350–1,500 at 120,000 resolution (at 200 m/z) with AGC set at 4 × 105 and maximum injection time of 50 ms. The most intense ions were selected for higher energy collision-induced dissociation at 38% collision energy with an isolation of 0.7 m/z, a resolution of 30,000, an AGC setting of 5 × 104 and a maximum injection time of 100 ms. Five of the 50 TMT batches were run on the Orbitrap Fusion mass spectrometer using the synchronous precursor selection-based (SPS)-MS3 method as previously described (Öztürk et al., 2022).

##### 2.1.1.3 TMT database searches and protein quantification

All RAW files (1,200 RAW files generated from 50 TMT 10-plexes) were analyzed using the Proteome Discoverer suite (v.2.3, Thermo Fisher Scientific). MS2 spectra were searched against the UniProtKB human proteome database containing both Swiss-Prot and TrEMBL human reference protein sequences (90,411 target sequences downloaded on 21 April 2015), plus 245 contaminant proteins. The Sequest HT search engine was used and parameters were specified as follows: fully tryptic specificity, maximum of two missed cleavages, minimum peptide length of six, fixed modifications for TMT tags on lysine residues and peptide N-termini (+229.162,932 Da) and carbamidomethylation of cysteine residues (+57.02146 Da), variable modifications for oxidation of methionine residues (+15.99492 Da) and deamidation of asparagine and glutamine (+0.984 Da), precursor mass tolerance of 20 ppm and a fragment mass tolerance of 0.05 Da for MS2 spectra collected in the Orbitrap (0.5 Da for the MS2 from the SPS-MS3 batches). Percolator was used to filter peptide spectral matches and peptides to an FDR <1%. Following spectral assignment, peptides were assembled into proteins and were further filtered based on the combined probabilities of their constituent peptides to a final FDR of 1%. In cases of redundancy, shared peptides were assigned to the protein sequence in adherence with the principles of parsimony. Reporter ions were quantified from MS2 or MS3 scans using an integration tolerance of 20 ppm with the most confident centroid setting.

#### 2.1.2 Mouse model data

The cohort of 40 mice used in our analysis contained 34 control mice and six APPSwDI/Nos2^−/−^ (CVN-AD) mice. The CVN-AD mouse model of AD used in our study expresses human APP with the Swedish-Dutch-Iowa mutations that are associated with early-onset AD in humans, and that cause the development of amyloid plaques in the brains of the mice, thereby corresponding to human AD. The CVN-AD mouse model was chosen for this study because it also possesses the Nos2 deletion, to better reflect the human immune response, unlike all other mouse models of AD [17–20]. The distribution of the control mice by genotype, age and sex is provided in Table 1. For this study, we used a set of control mice that covered several genetic backgrounds that have similarity to the backgrounds that are associated with AD risk in humans: that is: APOE genotype, age, sex and NOS2 gene expression. The use of a diverse set of control mice was used to provide a set of controls that would correspond more closely to the diversity of controls in the human sample. The statistical models were adjusted for the covariates of mouse genotype, age and sex. Peptides for both mouse Apoe and human Apoe were quantified. All mouse model proteomics data is included in Supplementary Table S1.

##### 2.1.2.1 Proteomics analysis for the mouse model data

###### Brain tissue preparation

Brain tissue samples stored in 1.5 mL tubes were delivered to the Duke Proteomics and Metabolomics Core Facility (*n* = 6 per genotype). 0.5% w/v ALS-1 surfactant in 50 mM ammonium bicarbonate (AmBic) was added to each sample at a volume of 10 uL/mg wet weight of tissue. Tissue homogenization and cell lysis was performed with probe sonication (Misonix) over three pulses at power level 3 for 5 s each with cooling on ice between pulses. A five uL aliquot of homogenate was diluted 25x in AmBic for determination of protein content by Bradford assay. Based on Bradford results, samples were 0.7 ± 0.2 mg protein/mg tissue. Following normalization (100 μg protein at 1 mg/mL protein in 0.5% ALS-1/AmBic), samples were reduced with 10 mM dithiothreitol (DTT) at 80°C with shaking for 15 min, alkylated with 20 mM iodoacetamide (IAA) at room temperature in the dark for 30 min, and digested with 2 μg sequencing grade modified trypsin (Promega) overnight at 37°C with shaking. Digestion was stopped with the addition of 12 μL 10/20/70 v/v/v TFA/MeCN/H2O and heating at 60°C for 2 h and diluted further with 1/2/97 v/v/v TFA/MeCN/H2O for a final digested protein concentration of 0.5 ug/uL. A pool of all samples (Study Pool QC, SPQC) was created from equal volumes of each sample, and analyzed at regular intervals throughout the study to allow observation of any experimental drift.

##### 2.1.2.2 Proteomics analysis

The samples were analyzed using a nanoAcquity UPLC system (Waters) coupled to a Q Exactive HF Orbitrap high-resolution accurate-mass tandem mass spectrometer (Thermo Scientific) via a nanoelectrospray ionization source. Each sample was analyzed once, and the SPQC was analyzed approximately every six samples. Briefly, the sample was first trapped and desalted on a Symmetry C18 180 um x 20 mm trapping column (5 uL/min at 99.8/0.1/0.1 v/v water/acetonitrile/formic acid), then the analytical separation was performed using a 1.7 um Acquity HSS T3 C18 75 um x 250 mm column (Waters). The peptides on the column were eluted using a 90-min gradient of 5%–40% acetonitrile with 0.1% formic acid at a flow rate of 400 nliters/min (nL/min) with a column temperature of 55°C. Data collection on the Q Exactive HF mass spectrometer was performed in a data-dependent MS/MS manner, using a 120,000 resolution precursor ion (MS1) scan followed by MS/MS (MS2) of the top 12 most abundant ions at 30,000 resolution. MS1 was accomplished using an automatic gain control (AGC) target of 3e6 ions and mass accumulation time of up to 50 msec. MS2 used AGC target of 5e4 ions, up to 45 msec maxiumum ion accumulation, 1.2 m/z isolation window, 27V normalized collision energy, and 20 s dynamic exclusion.

Following the analyses, the data was imported into Rosetta Elucidator v 4.0 (Rosetta Biosoftware, Inc.), and all LC-MS files were aligned based on the accurate mass and retention time of detection ions (“features”) using a PeakTeller algorithm (Elucidator). The relative peptide abundance was calculated based on area-under-the-curve (AUC) of aligned features across all runs. The MS/MS data was searched against a custom built database based on the SwissProt database with *Mus musculus* taxonomy (downloaded 28 April 2017) with additional proteins, including yeast ADH1_YEAST (surrogate standard), ALBU_BOVIN (contaminant), APOE_HUMAN (genetic substitution), and additional mutated proteins expressed in the mice with sequences provided by the investigators, were also included in the custom database. An equal number of reversed-sequence “decoys” were appended to this “forward” DB for false discovery rate determination. A total of 3,084 proteins were quantified, and 2,118 (69%) proteins were quantified with two or more peptides (Supplementary Table S1).

### 2.2 Statistical methods

#### 2.2.1 LASSO logistic regression

LASSO logistic regression is an adaptation of linear regression that uses shrinkage to reduce model complexity for binary classification problems (Tibshirani, 1997; Qian et al., 2020; Reisetter and Breheny, 2021). We use this as the baseline model for comparison with the Partial Least Squares method.

#### 2.2.2 Partial least squares

Partial Least Squares (PLS) is a generalization of multiple linear regression and is well suited for proteomic data analysis (Boulesteix and Strimmer, 2007) since it is designed to address the high correlations between the independent variables. It is a data reduction method that identifies specifically the variation of independent variables (X) that correlates with the output of interest (Y). In our model, the independent variable matrix (X) are the protein concentrations and baseline information of the samples controlling for age and sex. The response variable Y is a binary vector containing zeros and ones for whether the subject has AD (or expresses the causal APP variants in the case of the mouse model) or not.

When X are correlated rather than orthogonal, ordinary linear regression estimates can become unstable. PLS regression overcomes this collinearity problem by finding uncorrelated variables (i.e. principal component scores) and then uses multiple linear regression to regress the principal components (PC’s) against the Y, or the response variable. This allows PLS to provide substantial prediction results as well as having robust descriptive power. In contrast to multiple linear regression, which scales and offsets each variable in X as independent entities separately to model the output Y, PLS takes X as an entire matrix and iteratively transforms both X and Y matrices to maximize their covariance (Cramer, 1993). Because the response variable of interest for this study is a binary categorical variable (i.e. either the subject has AD or not), we used Partial least squares-discriminant analysis (PLS-DA), a variant of PLS, for this study.

PLS is not only good for predictive and descriptive modeling, but also for variable selection. Variable Importance in Projection (VIP) score, calculated based on loading scores from PLS results, estimates the relevance, and hence importance, of each variable in X in determining the response variables Y. Loading scores are weights that are estimated from the relationships between variables in the data for both the proteomics data matrix (X). Highly correlated variables have similar loading score. Therefore, VIP, based on these scores, can measure the importance of a gene with respect to both the response variable as well as the proteomics data matrix. Because the PLS properties of dimensionality reduction and variable selection are tightly related, multiple latent components are taken into account in the variable selection procedures, and hence the approach can discover non-linear patterns in the data (Boulesteix and Strimmer, 2007). In contrast to PLS-DA, LASSO regression does not model the covariation among independent variables.

Since an aim of this study is to identify and then analyze the proteins that are highly associated with the outcome of interest, specifically onset of AD, we used PLS-DA mainly as a gene selection approach utilizing the variable selection procedures, specifically the calculation of variance importance scores. The first step of the analysis identified the proteins that were significantly different between individuals with AD and cognitively normal in the human data (ROSMAP dorsolateral prefrontal cortex) and significantly different between the CVN-AD mice and control mice. As a second step, gene-set enrichment analysis was then performed on the resulting data. Finally, the individual protein results and gene set enrichment results were used to make the interspecies comparisons.

### 2.3 Gene set enrichment analysis

Gene set enrichment analysis (Subramanian et al., 2005) was carried out with the GENE2FUNC algorithm implemented in Functional Mapping and Annotation of Genome-Wide Association Studies (FUMA) version v1.3.8 (Watanabe et al., 2017). For the 20 input genes for the mouse and human datasets, the unique Entrez identification numbers were used in the analysis. All genes with an Entrez identification number (19,277) were used as the background gene set for the hypergeometric test. The Molecular Signatures Database v7.0 (August 2019) was used for the set of potential biological signatures. The Benjamini–Hochberg method was used as a correction for multiple testing with a maximum adjusted *p*-value of 0.05 for gene-set enrichment tests.

## 3 Results

### 3.1 Statistical model comparisons

First, we assessed the performance of the two statistical methods, LASSO logistic regression and PLS-DA on both mouse and human proteomics datasets. The accuracy scores of both methods are very similar for the mouse data (∼0.97). For the human data, however, the accuracy of the model using LASSO logistic regression is 0.63 while the accuracy of the model using PLS-DA method is 0.66. The difference in accuracy between the human and mouse datasets may be attributed to the fact that, excepting the sex chromosomes, the mice are genetically uniform, in contrast to the human subjects. Additionally, the mouse dataset is smaller, and hence potentially more focused, than the human dataset and can be modelled with fewer adjustable parameters than the human dataset.

### 3.2 Individual protein results for human and mouse

#### 3.2.1 LASSO regression

The top eight proteins identified by LASSO regression as significantly associated with carriage of the causal APP mutation for the mouse model are shown in Table 2. Table 3 summarizes the top eight proteins identified as significantly associated with Alzheimer’s disease risk in the human data. We will restrict our remarks to a few points for each case. In the mouse comparison, the protein with the highest beta coefficient is hexosaminidase B (Hexb), the beta-subunit of the lysosomal glycosyl hydrolase hexosaminidase. Hexosaminidase degrades molecules containing terminal N-acetyl hexosamines, many of which are related to the extracellular matrix. Deficits in Hexb in mice, and its ortholog HEXB in humans, are associated with lipid storage diseases and neurodegeneration that is accompanied by activated microglia. In contrast to the present study, in which Hexb expression in APP-expressing mice is elevated compared with control mice, Masuda et al. report that microglial Hexb expression is stable in a variety of neurodegenerative conditions in the mouse, including in the 5xFAD mouse (Masuda et al., 2020), which is another APP-expressing mouse line. The second highest expressed protein in the mouse comparison is Amyloid Precursor Protein (APP). This is not unexpected since the CVN-AD mouse is an APP transgenic model. In addition to serving as the precursor for amyloidogenic peptides, APP is a cell surface receptor that is involved in cellular adhesion and regulates neurite outgrowth and synaptogenesis (Müller and Zheng, 2012; Baumkötter et al., 2014). By contrast to these up-regulated genes, Neuronal Guanine Exchange Factor (Ngef), is down-regulated in the CVN-AD model, altering actin dynamics and disrupting growth cone motility (Shamah et al., 2001).

**TABLE 2:** Coefficients of important (by VIP scores) mouse genes with corresponding human genes identified from logistic LASSO regression results on the mouse proteomics data.

Mouse genes	Corresponding human genes	Coefficient
(intercept)	-	-57.227
APP	A4	0.781
CLU	CLUS	0.371
ENOPH	ENOPH	-0.783
HEXB	HEXB	2.234
HTRA	HTRA	0.416
LRP	LRP1	0.011
NGEF	NGEF	-1.521
MPST	THTM	0.226

**TABLE 3:** Top eight coefficients of important (by VIP scores) human genes from logistic LASSO regression results on the human data.

Human genes	Coefficient
(intercept)	−0.542
PDHX	−0.049
GHDC	0.306
COL23A1	−0.004
ALDH1A3	0.161
ANK2	1.671
GPC4	0.203
PDIA4	−0.268
ACTN4	−0.131

Methionine metabolism is critical for white matter synthesis and is defective in human AD (Linnebank et al., 2010; Hooshmand et al., 2019; Mihara et al., 2022) and in the CVN-AD mouse (Colton, CA, unpublished observations). Enolase-phosphatase 1 (Enoph1), which is highly expressed in stress responses of the brain (Wang et al., 2005; Fagerberg et al., 2014), is also involved in methionine salvage (Pirkov et al., 2008; Barth et al., 2014; Wang et al., 2021). Down regulation of this enzyme in this mouse model further implicates methionine disruption as a likely contributor to AD-like brain pathology.

In the human comparison, the top ranked protein is Ankryn (ANK2), which tethers integral membrane proteins to the underlying extracellular matrix. The second highest, GH3 Domain Containing (GHDC), may be involved in microtubule cytoskeleton organization and microvesicular trafficking, by analogy with the fly Dmel\TTL1A gene data (Janke et al., 2005). Its up-regulation may be in response to the formation of neurofibrillary tangles. The third highest ranked protein, Glypican (GPC4), is an integral membrane proteoglycan that is involved with the endosomal trafficking of ApoE-bound receptors and may itself be an ApoE receptor; it has been implicated as a cause of APOE4-dependent tau pathology (Saroja et al., 2022). Among other reactions, Aldehyde Dehydrogenase 1A3 (ALDH1A3), the fourth-highest ranked protein, catalyzes the conversion of retinal to all-trans retinoic acid (Moretti et al., 2016). Retinoic acid is the ligand for the RXR receptor, which, via heterodimerization with a number of other nuclear receptors (e.g., PPARa, PPARg, PPARd, LXR, FXR), regulates lipid and glucose metabolic pathways and the innate immune response (Saunders et al., 2021).

LASSO regression analysis also revealed that expression of Pyruvate Dehydrogenase Component X (PDHX) was reduced in AD. PDH is a multimeric intramitochondrial complex that generates acetyl-CoA from pyruvate, linking glycolysis with the TCA cycle and providing acetyl-CoA for neurotransmitter synthesis, epigenetic regulation and post-translational modification of proteins (Jankowska-Kulawy et al., 2022). PDH activity is reduced in AD (Bubber et al., 2005). PDHX couples the dihydrolipoamide dehydrogenase (E3) component of PDH to the central core subunit, dihydrolipoamide acetyltransferase (E2) (Škerlová et al., 2021). The activity of the overall complex is regulated by loosely associated PDH kinases and a PDH phosphatase. The latter is Ca^2+^-sensitive (Roche et al., 2001), and reduced PDH activity in AD has been attributed to altered mitochondrial calcium homeostasis (McCormack and Denton, 1989; Calvo-Rodriguez and Bacskai, 2021). The reduced PDHX expression may also be a contributing factor.

#### 3.2.2 PLS-DA

**FIGURE 1:**
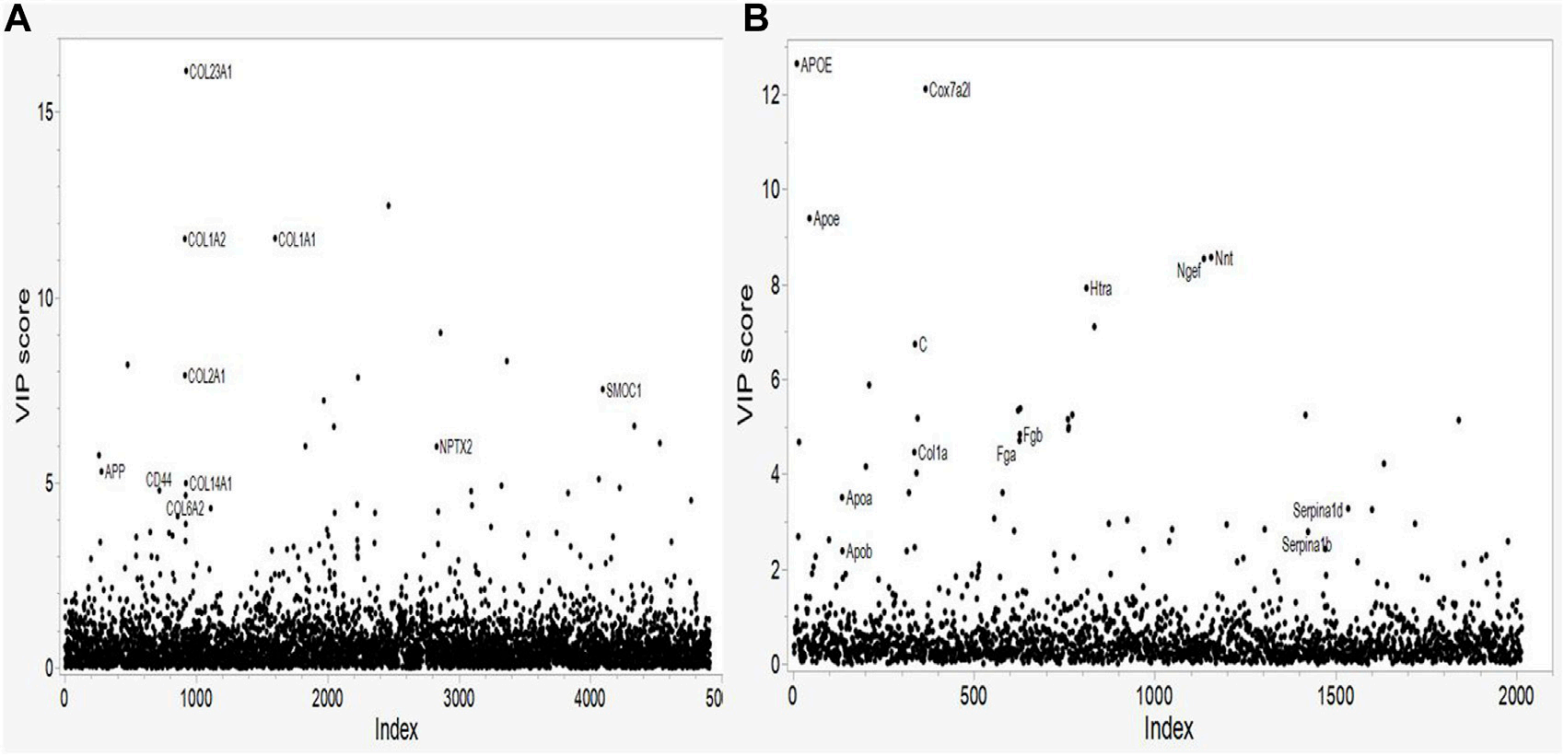
Variable Importance in Projection (VIP) plots from LASSO regression for **(A)** Human and **(B)** Mouse data. Predictors are the individual proteins. Labels on the plots represent the protein symbols.

The variance importance plots for all of the protein concentration data based on the PLS-DA model are shown in Figure 1. Relatively few proteins show VIP scores greater than 2.5 for either species. For the human proteomic data (Figure 1A) several collagens (COL1A1, COL1A2 and COL23A1) show VIP scores greater than 10; one collagen has a VIP of approximately 8 (COL2A1); two collagens (COL6A2 and COL14A1), the amyloid precursor protein (APP) and the CD44, NPTX2 and SMOC1 proteins have VIP scores of approximately 5. These proteins show the strongest effect on the human AD phenotype. For the mouse proteomics data (Figure 1B), the Apoe and Cox7A2L proteins show VIP scores greater than 9. Of note, the peptides that map to both the mouse apolipoprotein E protein (designated Apoe) and that map to the human ApoE protein (designated APOE) have VIP scores in the 8-13 range. Several proteins (Htra, C (complement), Nnt, Ngef, Fga, Fgb) show intermediate level VIP scores in the range of greater than 4.7 but less than 10. Interestingly, two other apolipoproteins, Apoa and Apob and two serpine proteins, Serpina1d and Serpina1b, show VIP scores of approximately 3. The collagen Col1a has a VIP score of approximately 5.

Peptides for both the mouse ApoE protein (designated Apoe) and human ApoE protein (designated APOE) were quantified, full results for all of the mouse models are provided in Supplementary Table S2. ApoE protein concentrations as measured by log2 (intensity) for the CVN and control mouse models are shown in Table 4. The human ApoE protein concentration is similar for the ApoE replacement mouse models, however, the concentration is lower in the CVN mouse and the two models that do not contain the ApoE replacement. The mouse ApoE levels are similar across the genotypes with the CVN mouse, HuNOS2 and NOS2 knock out mice showing slightly higher but similar concentrations.

**TABLE 4:** Protein intensities for the human and mouse ApoE proteins for the mouse model genotypes.

Genotype	N	APOE human log2 (intensity)	APOE mouse log2 (intensity)
ApoE-3	5	31.3 (0.25)	30.4 (0.38)
ApoE-4	5	30.8 (0.20)	29.9 (0.24)
CVN	6	26.0 (0.11)	33.3 (0.23)
E3 HN	6	31.1 (0.13)	30.2 (0.08)
E4HN	6	30.8 (0.07)	29.9 (0.09)
HuNOS2	6	26.2 (0.24)	32.4 (0.12)
NOS2 KO	6	26.2 (0.14)	32.5 (0.06)

The PLS-DA model enables interpretation of the magnitude and direction of the difference in levels of the phenotype (CVN-AD vs. control mice, AD vs. cognitively normal humans) in context of linear and logistic regression models. Table 5 presents the top 20 proteins, based on VIP scores, that show differences in concentration between individuals with LOAD in contrast to cognitively normal individuals, and between the CVN-AD and control mouse models. The direction of the effect for the beta coefficient is positive for the CVN-AD model compared with control mice and, for the human data, for the AD samples relative to cognitively normal controls (Table 4). Positive coefficients show that the protein concentration is estimated to be higher in the CVN-AD mouse model or in human samples with AD.

**TABLE 5:** The top 20 human and mouse genes from the PLS-DA’s VIP results.

Human	Coefficient	SE	Mouse	Coefficient	SE
COL23A1	0.0476	0.015	APOE	0.036	0.0159
MDK	−0.036	0.0108	Nnt	0.0244	0.0128
COL1A1	0.0343	0.012	Ngef	0.0215	0.0104
COL1A2	0.0342	0.012	Igh	0.0215	0.0093
NTN1	−0.0267	0.0076	C	0.019	0.0073
PRPH	−0.0245	0.0087	Ahsg	0.0161	0.0075
BGN	0.0242	0.0087	Pzp	0.0155	0.0045
COL2A1	0.0115	0.0096	Hba	0.0149	0.0046
KRT86	0.0231	0.0078	Fgg	0.0143	0.0043
SMOC1	−0.0222	0.0056	Hbb.b.1	0.0139	0.0042
HP	−0.0213	0.0074	Cox7a21	−0.028	0.0169
TAGLN	0.0193	0.007	G1b	0.0132	0.0014
IGHA1	−0.0192	0.0079	Hbb.b.1	0.013	0.0038
TPM2	0.0179	0.0062	Fga	0.012	0.0046
GSTM1	0.0177	0.015	Fgb	0.0113	0.0043
NPTX2	0.0176	0.0034	Hebp	0.0112	0.006
APCS	−0.0169	0.005	Mpst	−0.0128	0.006
APP	−0.0156	0.0054	Pomc	−0.0131	0.0231
SLC38A2	−0.015	0.0037	Htra	−0.0206	0.0095
COL14A1	0.0147	0.0058	Apoe	−0.0254	0.0114

### 3.3 Gene set enrichment results

Gene set enrichment analysis was performed for the human and mouse data separately using the 20 genes in the respective human and mouse sets with the strongest signals defined by VIP scores (Table 5).

**FIGURE 2:**
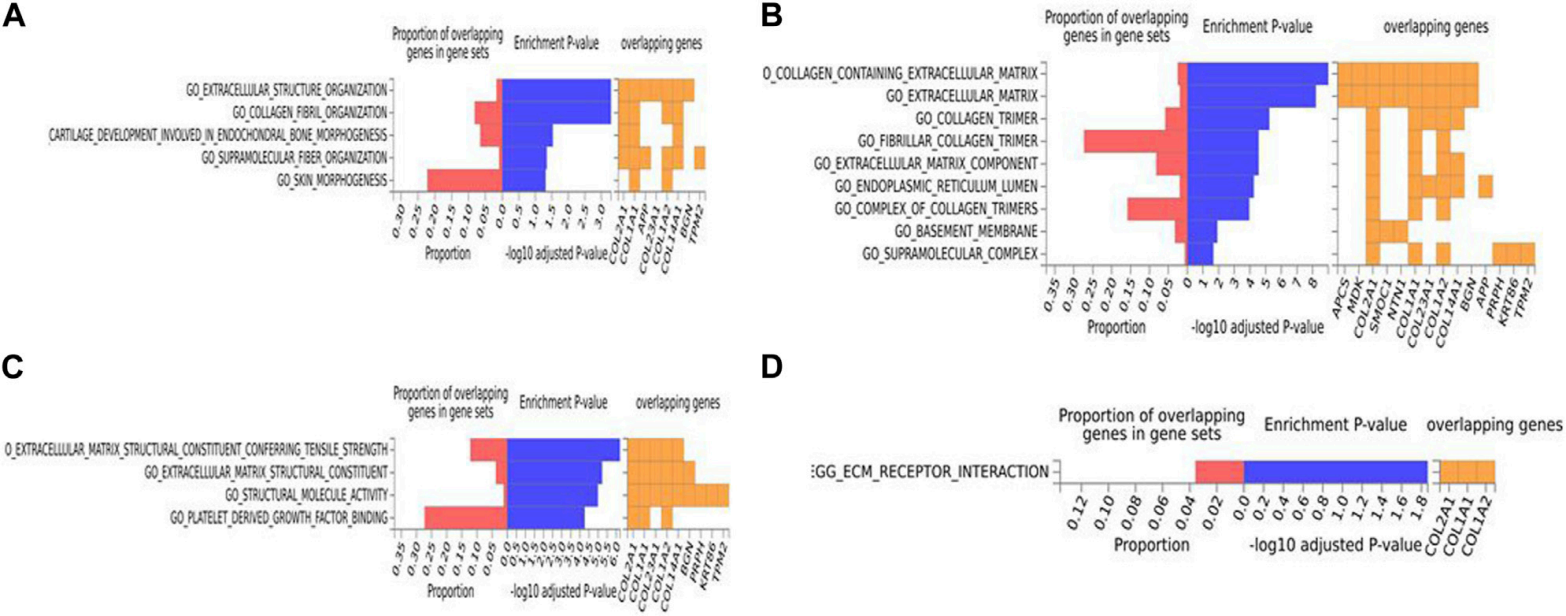
Biological pathway enrichment analysis for the human data. The top 20 proteins identified from the differential protein abundance analysis were used as the input dataset. Each plot shows the proportions of overlapping proteins (proteins that overlap with the proteins in the specific gene set list), - log10 of the enrichment *p*-value (from the hypergeometric test, adjusted for false discovery rate) and identity of proteins that are overlapping with the tested gene sets. The panels are derived for each of the Gene Ontology (GO) gene sets/pathways Reactome or KEGG pathway database. **(A)** GO Biological Functions, **(B)** GO Cellular Components, **(C)** GO Molecular Functions, **(D)** KEGG.

**FIGURE 3:**
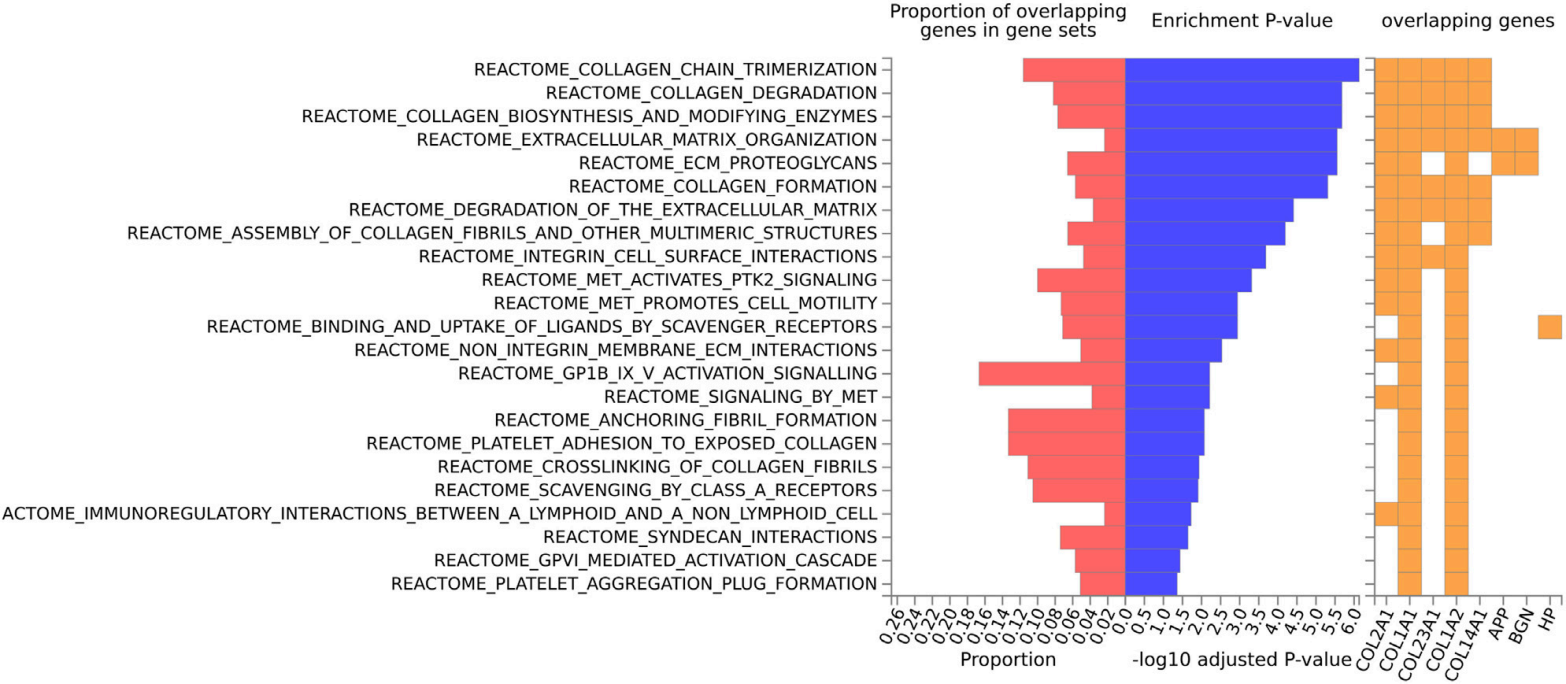
Biological pathway enrichment analysis for the human data for the Reactome pathway database. The top 20 proteins identified from the differential protein abundance analysis were used as the input dataset. The plot shows the proportions of overlapping proteins (proteins that overlap with the proteins in the specific gene set list), - log10 of the enrichment *p*-value (from the hypergeometric test, adjusted for false discovery rate) and identity of proteins that are overlapping with the tested gene sets.

For the human data (Figures 2, 3), under GO biological processes (Figure 2A) and molecular function (Figure 2C), FDR (false discovery rate)-significant pathways included collagen, fibrils and the extracellular matrix (ECM). FDR-significant reactome (Figure 3) pathways included ECM, collagen and integrins. The only FDR significant pathway for KEGG (Figure 2D) is the extracellular matrix-receptor interaction.

**FIGURE 4:**
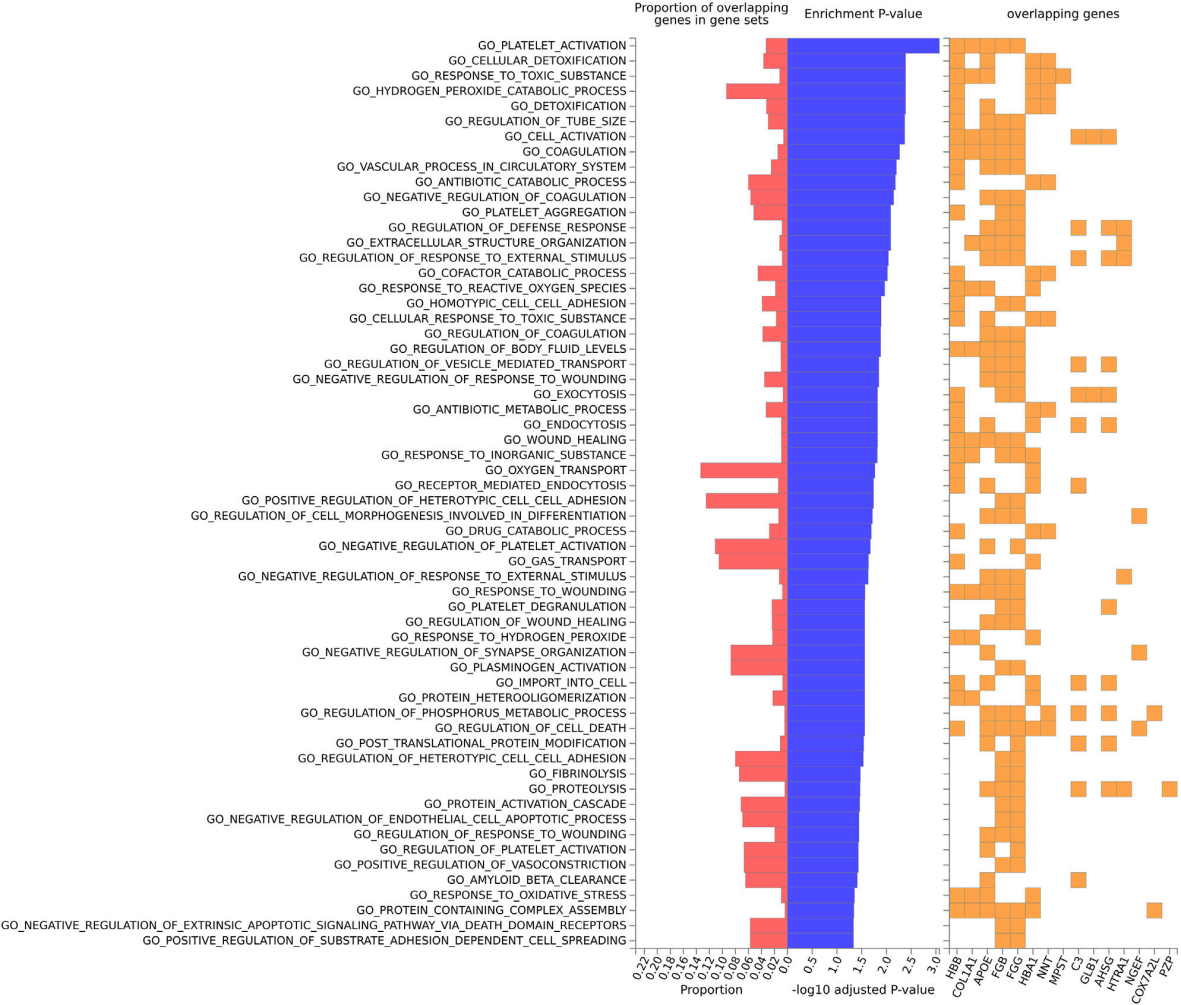
Biological pathway enrichment analysis for the mouse model data for the GO Biological Function database. The top 20 proteins identified from the differential protein abundance analysis were used as the input dataset. The plot shows the proportions of overlapping proteins (proteins that overlap with the proteins in the specific gene set list), - log10 of the enrichment *p*-value (from the hypergeometric test, adjusted for false discovery rate) and identity of proteins that are overlapping with the tested gene sets.

**FIGURE 5:**
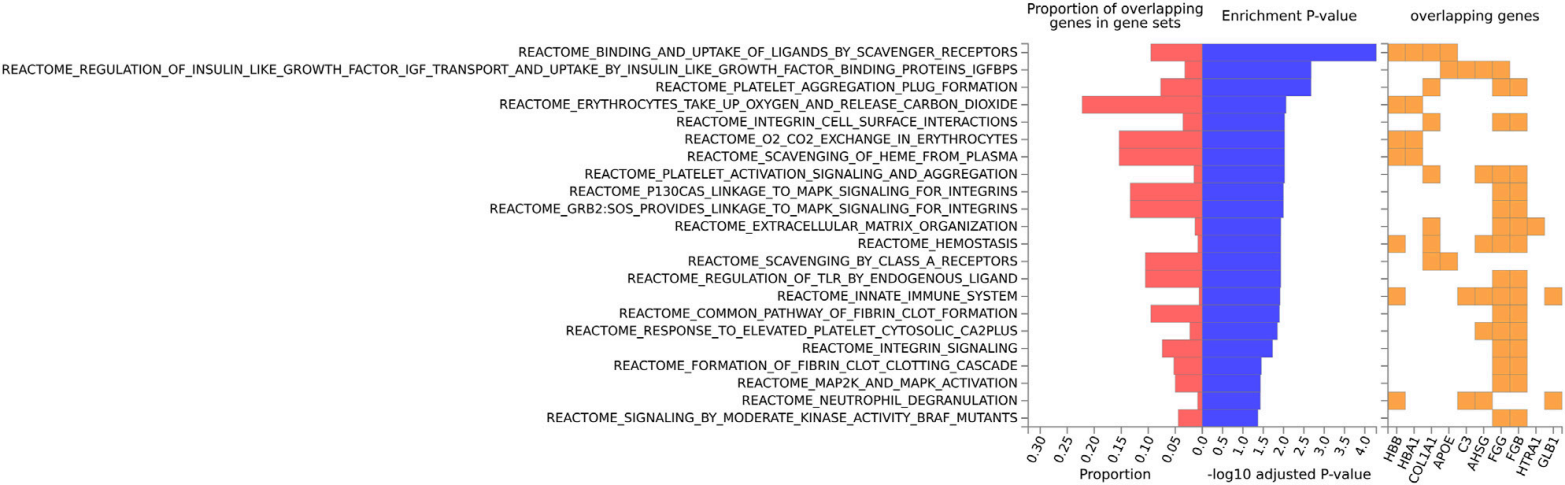
Biological pathway enrichment analysis for the mouse data for the Reactome pathway database. The top 20 proteins identified from the differential protein abundance analysis were used as the input dataset. The plot shows the proportions of overlapping proteins (proteins that overlap with the proteins in the specific gene set list), - log10 of the enrichment *p*-value (from the hypergeometric test, adjusted for false discovery rate) and identity of proteins that are overlapping with the tested gene sets.

**FIGURE 6:**
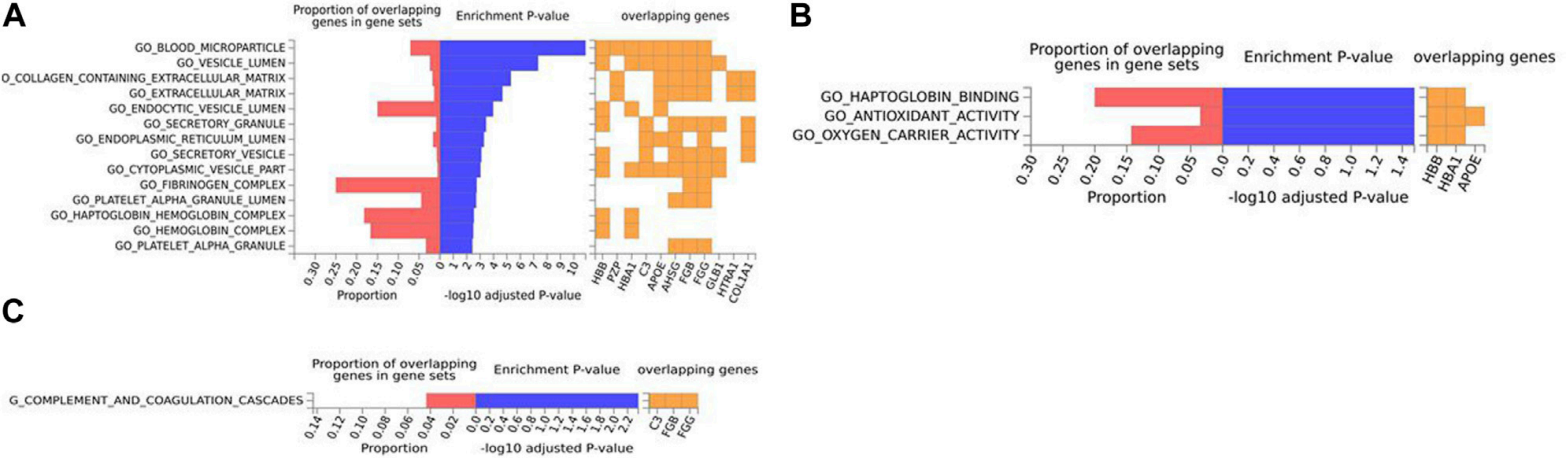
Biological pathway enrichment analysis for the mouse model data. The top 20 proteins identified from the differential protein abundance analysis were used as the input dataset. The plot shows the proportions of overlapping proteins (proteins that overlap with the proteins in the specific gene set list), - log10 of the enrichment *p*-value (from the hypergeometric test, adjusted for false discovery rate) and identity of proteins that are overlapping with the tested gene sets. The panels are derived for each of the Gene Ontology (GO) gene sets/pathways Reactome or KEGG pathway database. **(A)** GO Cellular Components, **(B)** GO Molecular Functions, **(C)** KEGG.

For the mouse data (Figures 4–6), strong GO biological processes (Figure 4) signals were observed for reactive oxygen species, cell adhesion/coagulation, endocytosis, amyloid beta clearance and cell death. For the reactome (Figure 5), FDR-significant pathways included: innate immunity, complement and coagulation and integrins. The only FDR significant pathway for KEGG (Figure 6C) is complement and coagulation cascades.

Pathway signatures that showed FDR-adjusted *p* values ≤0.05 for both the human and mouse datasets are shown in Table 6. It is important to highlight that extracellular matrix pathways and integrin-related pathways were dysregulated in both the human and mouse data.

**TABLE 6:** Comparison of human and mouse model signatures.

GeneSet of human signatures	Category of human signatures	adjP of human signatures	adjP of mouse signatures
GO_EXTRACELLULAR_STRUCTURE_ORGANIZATION	GO_bp	5.10E-04	8.22E-03
GO_COLLAGEN_CONTAINING_EXTRACELLULAR_MATRIX	GO_cc	1.01E-09	5.02E-06
GO_EXTRACELLULAR_MATRIX	GO_cc	6.21E-09	2.14E-05
GO_ENDOPLASMIC_RETICULUM_LUMEN	GO_cc	5.69E-05	5.20E-04
REACTOME_EXTRACELLULAR_MATRIX_ORGANIZATION	Curated_gene_sets	7.30E-06	1.68E-02
PID_INTEGRIN1_PATHWAY	Curated_gene_sets	1.84E-04	8.17E-03
REACTOME_INTEGRIN_CELL_SURFACE_INTERACTIONS	Curated_gene_sets	3.40E-04	1.11E-02
REACTOME_BINDING_AND_UPTAKE_OF_LIGANDS_BY_SCAVENGER_RECEPTORS	Curated_gene_sets	1.73E-03	9.56E-05
REACTOME_EXTRACELLULAR_MATRIX_ORGANIZATION	Reactome	2.78E-06	1.68E-02
REACTOME_INTEGRIN_CELL_SURFACE_INTERACTIONS	Reactome	2.06E-04	7.37E-03
REACTOME_BINDING_AND_UPTAKE_OF_LIGANDS_BY_SCAVENGER_RECEPTORS	Reactome	1.14E-03	9.56E-05
REACTOME_SCAVENGING_BY_CLASS_A_RECEPTORS	Reactome	1.23E-02	1.94E-02
REACTOME_PLATELET_AGGREGATION_PLUG_FORMATION	Reactome	4.35E-02	2.90E-03

GeneSet of human signatures	Genes of human signatures	Genes of mouse signatures
GO_EXTRACELLULAR_STRUCTURE_ORGANIZATION	COL2A1:COL1A1:APP:COL23A1:COL1A2:COL14A1:BGN	HTRA1:COL1A1:APOE:FGB:FGG
GO_COLLAGEN_CONTAINING_EXTRACELLULAR_MATRIX	APCS:MDK:COL2A1:SMOC1:NTN1:COL1A1:COL23A1:COL1A2:COL14A1:BGN	HTRA1:PZP:COL1A1:APOE:AHSG:FGB:FGG
GO_EXTRACELLULAR_MATRIX	APCS:MDK:COL2A1:SMOC1:NTN1:COL1A1:COL23A1:COL1A2:COL14A1:BGN	HTRA1:PZP:COL1A1:APOE:AHSG:FGB:FGG
GO_ENDOPLASMIC_RETICULUM_LUMEN	COL2A1:COL1A1:APP:COL23A1:COL1A2:COL14A1	COL1A1:C3:APOE:AHSG:FGG
REACTOME_EXTRACELLULAR_MATRIX_ORGANIZATION	COL2A1:COL1A1:APP:COL23A1:COL1A2:COL14A1:BGN	HTRA1:COL1A1:FGB:FGG
PID_INTEGRIN1_PATHWAY	MDK:COL2A1:COL1A1:COL1A2	COL1A1:FGB:FGG
REACTOME_INTEGRIN_CELL_SURFACE_INTERACTIONS	COL2A1:COL1A1:COL23A1:COL1A2	COL1A1:FGB:FGG
REACTOME_BINDING_AND_UPTAKE_OF_LIGANDS_BY_SCAVENGER_RECEPTORS	HP:COL1A1:COL1A2	HBB:HBA1:COL1A1:APOE
REACTOME_EXTRACELLULAR_MATRIX_ORGANIZATION	COL2A1:COL1A1:APP:COL23A1:COL1A2:COL14A1:BGN	HTRA1:COL1A1:FGB:FGG
REACTOME_INTEGRIN_CELL_SURFACE_INTERACTIONS	COL2A1:COL1A1:COL23A1:COL1A2	COL1A1:FGB:FGG
REACTOME_BINDING_AND_UPTAKE_OF_LIGANDS_BY_SCAVENGER_RECEPTORS	HP:COL1A1:COL1A2	HBB:HBA1:COL1A1:APOE
REACTOME_SCAVENGING_BY_CLASS_A_RECEPTORS	COL1A1:COL1A2	COL1A1:APOE
REACTOME_PLATELET_AGGREGATION_PLUG_FORMATION	COL1A1:COL1A2	COL1A1:FGB:FGG

## 4 Discussion

Our study focused on statistical approaches to reduce the dimensionality and address the collinearity of “omic” data, specifically proteomic data, in order to compare and contrast across species at the level of individual proteins and biological pathways. Proteomic samples obtained from individuals diagnosed with Alzheimer’s disease and controls were compared with samples from mouse models of AD where the contrast was between mice with a genetic mutation that accelerates the development of AD-related neuropathology and control mice. There were two aims to this study; first to compare statistical methods for addressing the collinearity and high dimensionality of the data for cross-species comparisons, and second to assess the species differences and similarities at the protein and pathway levels. We completed a comprehensive comparison of LASSO regression and Partial Least squares discriminant analysis (PLS-DA) for the analysis of multivariate, high dimensionality datasets with high collinearity. The PLS-DA provided the better statistical performance. The major biological finding of the study was that extracellular matrix proteins and integrin-related pathways were dysregulated in both the human and mouse data. These findings were observable at both the individual protein and pathway levels. The signals in the CVN-AD model that were related to reactive oxygen species (ROS) and innate immunity may reflect adjustments made by the mouse genome to accommodate the loss of Nos2, which is central to both the innate immune response and the generation of ROS species. Likewise, the signal in amyloid clearance could reflect adjustments to the elevated levels of amyloid precursor protein expression in this model, which is estimated to be ca. 1.5X the normal level because it expresses both the human form, at ∼0.5X the mouse level, and mouse App.

The morphogenesis of the CNS and the successful differentiation of all the cell types within it depend on regulatory interactions between the cells and their environments. The extracellular matrix plays an essential role in this communication, and is involved in bidirectional signaling, in-out as well as out-in, from guiding cells and axons during the elaborate processes of developing nerve connections to maintaining tissue homeostasis and regulating cell function, based on ‘nearest neighbors’ signaling. Prior research has suggested the involvement of extracellular matrix (ECM) and integrins in the physiological processes involved in the development of AD. A recent review provided details on the specific ECM proteins that are modulated in the neuropathology of AD (58). Interestingly, the ECM has roles both in regulation of beta amyloid through modulation of amyloid precursor protein (Small et al., 1993; Beyreuther et al., 1996; Ma et al., 2020) and neuroprotection (Cheng et al., 2009; Conejero-Goldberg et al., 2014; Suttkus et al., 2016). ECM substrates fibronectin and vitronectin, but not laminin, promote microglial activation and increased expression of several integrins, cytokines and ECM that are involved in regulation of microglial activity (Milner and Campbell, 2003).

The extracellular matrix (ECM) is comprised of numerous cellular components including proteoglycans, glycosaminoglycans, proteins, proteinases, and cytokines. ECM components are synthesized by both neurons and astrocytes and play an important role in the formation, maintenance, and function of synapses in the central nervous system (CNS) (Dzyubenko et al., 2016). In the CNS, the ECM contains the basement membrane (basal lamina), perineuronal nets, and the neural interstitial matrix (Lau et al., 2013; Mouw et al., 2014; Ma et al., 2020). The ECM is intimately involved in the regulation of beta amyloid. Elastin and heparan sulfate proteoglycans are involved in the upregulation of extracellular Ab. Collagen VI and laminin have been shown to interact with Ab peptides, possibly having an effect on Ab clearance. Our results from the human data show that numerous collagen proteins in addition to amyloid precursor protein are differentially expressed in AD brains in contrast to cognitively normal. Consistent with prior reports, the positive regression coefficients show that concentrations of the collagen proteins were upregulated in the human AD samples relative to cognitively normal (Kalaria and Pax, 1995; van Horssen et al., 2002; Bourasset et al., 2009; Cheng et al., 2009; Tong et al., 2010).

Prior evidence supports the involvement of integrin signaling pathways in the development of AD (58). Studies have suggested the involvement of both the ECM proteins and integrins in modulation of neuroplasticity (Chelyshev et al., 2022), synapse formation (Park and Goda, 2016) and axon regeneration (Pfundstein et al., 2022). It has been suggested that integrins undergo plasticity including clustering through interactions with ECM proteins, modulating ion channels, intracellular calcium and protein kinases signaling, and reorganization of cytoskeletal filaments (Wu and Reddy, 2012). Integrins are also involved in regulation of synapse formation, working with glial signals and neurotransmitter receptor dynamics to regulate synaptic plasticity (Park and Goda, 2016). Integrins also interact with the amyloid precursor protein (APP) (Pfundstein et al., 2022). APP regulates integrin-mediated adhesion and β1-integrins in turn regulate the processing of APP.

This study focused exclusively on analysis of proteomics data. This is in contrast to studies that focus on analysis of mRNA data, either from bulk brain tissue or single cell analysis. There are also studies that have analyzed both proteomics and multilayered omics data. Key findings from one recent study included identification of modules including MAPK/metabolism and matrisome that were associated with AD neuropathology (Johnson et al., 2022). The matrisome module was influenced by the APOE ε4 allele but was not related to the rate of cognitive decline after adjustment for neuropathology (Johnson et al., 2022). The MAPK/metabolism module was strongly associated with the rate of cognitive decline (Johnson et al., 2022). Relevant to our study, the matrisome module consists of a collection of ECM-associated proteins and glycosaminoglycan-binding proteins.

Our study has several strengths. First, two alternative statistical methods for addressing the high collinearity of the proteomic measures were compared with results from each approach reported. Multi-collinearity and dimensionality reduction are common issues for omics studies and this study addressed the question in context of cross-species analysis. For the pathway/signature analysis, well established databases including GO and reactome were used to enable replication studies and other future work. We used a mouse model that reflects the human innate immune response and that leads to age-dependent tau pathology and neuron loss. Careful mapping of mouse to human protein nomenclatures was performed for the proteomics results to allow cross-species comparison. For the mouse model, proteomic determination of both mouse and human APOE concentrations based on specific peptides was performed.

Other statistical methods that address translation between AD mouse models and human data, primarily transcriptomic data, have been published. Some of these approaches share similar a similar statistical basis to our study. Lee et al. presented an approach, “Translatable Components Regression” (Brubaker et al., 2020) that concurrently analyzed transcriptomic data from human brain and AD mouse models to identify pathway-level signatures present in the human data that were predictive of mouse model disease status (Lee et al., 2021). For this approach a principal component analysis (PCA) space for human data is derived and projected in a mouse dataset (Lee et al., 2021). Importantly, this work also utilized linear models to differentiate disease-specific effects from aging and demonstrated that the analysis framework identified cross-species signatures that do not necessarily dominate in at least one of the datasets separately (Lee et al., 2021). Other approaches have focused on cross-species gene set analysis (Miller et al., 2010; Burns et al., 2015), network analysis (Zhang et al., 2013; Mostafavi et al., 2018; Bai et al., 2020; Wang et al., 2020) and meta analyses of co-expression data (Friedman et al., 2018; Wan et al., 2020b). Of particular note are approaches that utilize ultra-deep level proteomics analysis coupled with integrated systems-biology analysis (Bai et al., 2020; Wang et al., 2020; Bai et al., 2021).

Our study also has several limitations. The sample size for the mouse study is relatively small; a larger sample would increase statistical power to detect differences between the CVN-AD and control mice. The comparison between the mouse and human results at both the individual protein and pathway levels are based on single datasets with comparisons to prior literature for the statistically significant proteins and pathways. Future studies could be planned to replicate the mouse, human and combined results using independent datasets. Finally, we used empirical thresholds of 20 proteins for inclusion in the pathway analysis and an FDR significance level of 0.05 for selection of pathways. Alternative analytical approaches and different thresholds may provide additional insight about these datasets.

Future work will assess the impact of age, sex and APOE genotype on the within-species and cross-species comparisons using approaches including the gene set enrichment likelihood ratio test which quantifies gene set enrichment accounting for covariate effects at the gene set level (Bryan et al., 2021), and it will involve additional mouse models of AD that incorporate known genetic risk factors, such as APOE4, and that are created by targeted replacement of the endogenous mouse gene with the hetrospecific isofunctional human homolog, to avoid potential non-specific effects that can blur transgenetic manipulations. A high research priority of the NIH is the development of improved mouse models of Alzheimer’s disease to improve reproducibility, transparency and translatability (https://www.model-ad.org/), and a number of new models have been developed (https://www.alzforum.org/news/research-news/cornucopia-loads-new-mouse-models-available). To identify the most translatable models, comparisons with human data bases, as we have done, will be essential. The intentional incorporation of alterations to the ECM and integrins along the lines discovered here might be a useful approach. In any event, the application of methods we developed here will be helpful in guiding new model development.

In summary, this study addressed several of the critical issues involved in cross-species comparisons of omic data, specifically proteomic data. In addition to providing guidance on alternative statistical approaches to analyze the data, the approach may help inform the development of mouse models that are more relevant to the study of human late-onset Alzheimer’s disease and provide insight about specific biological pathways identified as differentially regulated in individuals with AD and in AD mouse models. The biological results from the cross-species analysis point to specific protein targets that involve the extracellular matrix and integrin pathways. These results can be used to plan future, focused studies on longitudinal changes of these proteins and pathways in context of the development of Alzheimer’s Disease.

## Data Availability

The datasets presented in this study can be found in online repositories. The names of the repository/repositories and accession number(s) can be found in the article/Supplementary Material. The raw proteomics data has been uploaded to the MassIVE repository and can be downloaded at: ftp://massive.ucsd.edu/MSV000092255/). ROSMAP resources can be requested at: https://www.radc.rush.edu.

## Supplementary material

Supplementary Table S1 Proteomics data and sample information for the mouse models.

Supplementary Table S2 APOE peptide data for all of the mouse models.
